# Development of a complex palliative care intervention for patients with heart failure and their family carers: a theory of change approach

**DOI:** 10.1186/s12904-025-01776-5

**Published:** 2025-05-06

**Authors:** Bader Nael Remawi, Nancy Preston, Amy Gadoud

**Affiliations:** 1 Lancaster Medical School, Lancaster University, Lancaster, LA1 4AT UK; 2 Doctor of Pharmacy Department, Birzeit University, Birzeit, Palestine; 3 Division of Health Research, Lancaster University, Lancaster, LA1 4AT UK

**Keywords:** Palliative care, Heart failure, Complex intervention, Co-design, Normalisation process theory, Theory of change

## Abstract

**Background:**

Patients with heart failure have significant palliative care needs but few receive palliative care. Guidance is lacking on how to integrate palliative care into standard heart failure care. Palliative care interventions often lack an underpinning theory and details on how key components interact to achieve an impact. Understanding how and why an intervention works enhances implementation. This study aimed to develop and refine a theory-based, complex palliative care intervention for patients with heart failure and their family carers.

**Methods:**

A preliminary theory that underlies the intervention and delineates its key components was co-designed, based upon a literature review, in three Theory of Change workshops with stakeholders from a hospital heart failure multidisciplinary team. The workshop discussions and analysis were informed by Normalisation Process Theory. Subsequently, analysis of secondary data on patient and carer experiences with palliative care services was presented to stakeholders to refine the proposed theory. Service users were consulted to provide feedback on the intervention components.

**Results:**

The agreed impact of the intervention was to meet the holistic palliative care needs of patients with heart failure and their families. Three long-term outcomes were identified: reduced unnecessary hospitalisations, symptom burden, and caregiving burden. Twelve preconditions on the patient, family, and healthcare professional levels and contextual assumptions were determined to achieve these outcomes. Proposed intervention activities include educating patients and heart failure teams on palliative care, completing a needs-assessment tool (NAT: PD-HF), addressing primary palliative care needs, sharing a summary of the tool with healthcare staff, and sharing experiences of using NAT: PD-HF in practice.

**Conclusions:**

The study provided novel insights into complex intervention development and the potential mechanism of integrating palliative care in heart failure. It outlined how the complex intervention could work and identified the active ingredients necessary for replication. The developed Theory of Change serves as a model for researchers and policymakers to use in heart failure, but also as an example of how to develop interventions embedded in and co-produced from practice.

**Supplementary Information:**

The online version contains supplementary material available at 10.1186/s12904-025-01776-5.

## Background

Heart failure is a progressive disease that is associated with poor prognosis [1]. It is the most common diagnosis in patients aged 65 years or older admitted to hospitals in developed countries [2]. Heart failure causes a significant physical, psychological, social, and financial burden that is comparable to that of cancer [3, 4]. It also has an impact on family carers who may prioritise the health of their relative over their health [5, 6]. Most palliative care needs of patients with heart failure can be addressed by the cardiology team [7, 8]. For more complex needs, a specialist palliative care team is consulted. Integrating palliative care in heart failure minimises patient suffering and reduces the caregiving burden on family carers [5, 9]. Despite these benefits and guidelines’ call to integrate palliative care into standard heart failure care [10–13], most patients with heart failure have limited or late access to palliative care [14, 15]. Several barriers to providing palliative care to patients with heart failure exist, including the unpredictable illness trajectory, poor communication with patients and families about their condition, poor knowledge and attitude about palliative care, poor collaboration between healthcare professionals, and lack of resources [16].

Given these challenges, guidance is lacking on how best to integrate palliative care into standard heart failure care [9, 17]. Integrating palliative care in heart failure requires a complex, multi-component, and adaptable intervention [18]. Previous palliative care interventions in heart failure are often poorly described and lack guidance on how to implement palliative care in routine clinical practice [19]. The focus was on whether or not the interventions achieved specific outcomes, rather than how or why they achieved or failed to achieve them. Understanding how and why an intervention works in a specific context can identify possible barriers and resource constraints and enhance implementation [18, 20]. Detailed intervention description is also important to improve transparency, replicability, and implementation [20–22].

To develop feasible and implementable interventions, several guidelines call for using a programme theory to explore the active ingredients within complex interventions, causal mechanisms or pathways of change, and contextual factors [20, 22, 23]. When applied prospectively in the intervention development stage, programme theories trigger thinking about the choice and rationale of the intervention components and outcomes, hypothetical mechanism of action through which the intervention components achieve those outcomes, and possible implementation barriers [24]. Previous palliative care interventions, especially those for heart failure, mostly lacked an underpinning theoretical framework and a clear understanding of their effective components and mechanisms of action [18]. Detailed and systematic intervention development and a clear theoretical understanding of how the intervention might cause change are needed to develop effective interventions and identify weak links in the causal pathway [20].

This study aimed to develop a theory-based, complex palliative care intervention for patients with heart failure and their family carers. Specific objectives were:

- Develop a programme theory that underpins the intervention to show how and why it is expected to work.
- Represent the programme theory in a map to illustrate the hypothetical causal pathway of the intervention and mechanism of integrating palliative into standard heart failure care.
- Identify the ultimate impact (desired goal) of the intervention and the long-term outcomes that the intervention can achieve on its own.
- Identify the intermediate and short-term outcomes (preconditions) of the intervention and the intervention activities required to achieve the outcomes.
- Identify the rationales for the links in the hypothetical causal pathway and the contextual assumptions that must exist to achieve the outcomes.

## Methods

### Frameworks for intervention development

The intervention was co-produced with service provider and service user stakeholders using three complementary guides: the Medical Research Council (MRC) Framework for the Development and Evaluation of Complex Health Interventions [20], Bleijenberg et al.’s guidance on intervention design [22], and the Methods of Researching End of Life Care (MORECare) guidance [23]. Steps of the intervention development included identifying the defining the problem of integrating palliative care into standard heart failure care, examining current practice and context, determining the needs of service providers (healthcare professionals) and users (patients and families), identifying relevant existing evidence of palliative care integration in heart failure, developing a programme theory to understand how the intervention is expected to work, modelling process and outcomes and delineating the key intervention components, and describing the intervention design. More details about the steps of the intervention development are shown in Additional File 1.

### Theory of change development

In response to the frameworks for intervention development, the Theory of Change approach was developed to underpin our intervention [24]. Theory of Change is a theory of how and why a programme works and what the key components are. In this study as in similar palliative care studies [25–29], the resulting Theory of Change was developed with stakeholders and represented graphically in a Theory of Change map that illustrates the mechanisms of change and hypothetical causal pathways through which the intervention components interact within a specific context to achieve specific outcomes and a realistic impact. As part of the development of the Theory of Change, an intervention was defined and co-designed with stakeholders to enhance its effectiveness, implementation, and sustainability in practice.

The Aspen Institute’s and De Silva et al.’s practical guides to Theory of Change development were followed [30, 31]. The Theory of Change map was developed using a backwards mapping approach, starting by identifying the ultimate *impact* of the intervention that stakeholders hope to attain, then working backwards to identify the *long-term outcomes* and *preconditions* necessary to achieve that impact. Other steps included identifying the *interventions* required to achieve the outcomes and preconditions, the *rationales* for the links in the causal pathway, and the *assumptions* (contextual conditions) necessary to achieve the outcomes.

**Fig. 1:**
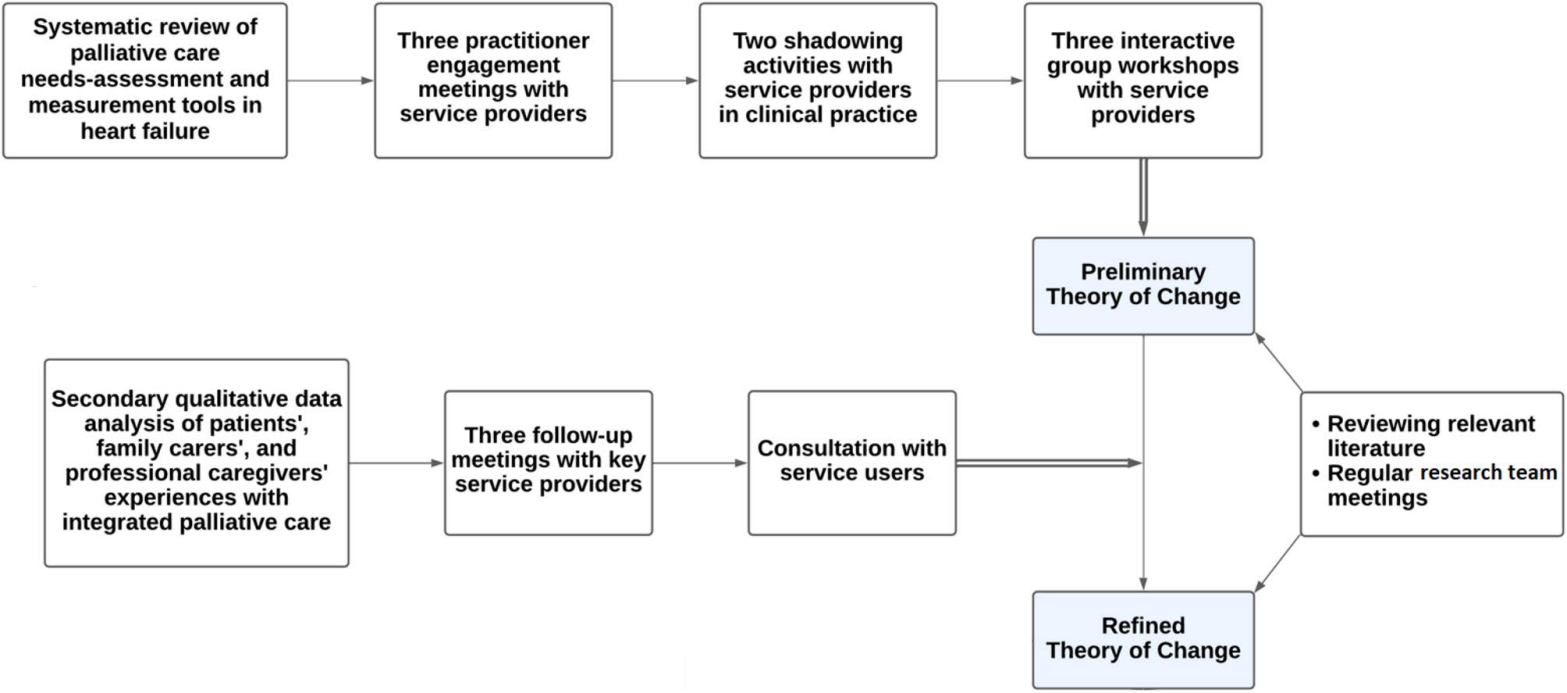
Stepwise development of the Theory of Change underpinning the intervention

The steps for the development of the Theory of Change underpinning the intervention are illustrated in Fig. 1. As preparatory work, practitioner engagement meetings with service providers, shadowing a key service provider in clinical practice, and presenting the findings of our systematic review of palliative care needs-assessment tools in heart failure [32] provided the necessary background and contextual data which informed the development of the Theory of Change. Next, a preliminary Theory of Change underpinning the intervention was co-produced incrementally over a series of workshops with service providers (see Data collection). Findings from our secondary data analysis of the experiences of advanced heart failure patients, family carers, and healthcare professionals with palliative care services [6] were then discussed in follow-up meetings with key service providers to refine the proposed theory. These data were collected from a multinational European project aiming to evaluate the perspectives of patients with advanced heart failure, cancer, and chronic obstructive pulmonary disease; family carers; and professional caregivers on integrated palliative care [33]. Subsequently, a Patient and Public Involvement (PPI) group was consulted which helped further refine the theory.

### Data collection

#### Theory of change workshops with service providers

Workshop participants were recruited from the heart failure multidisciplinary team of a tertiary teaching NHS hospital in a coastal community area in northwest England. The team oversees patients typically being managed medically. Three in-person group workshops were conducted between October 2019 and January 2020. Four to eight service providers participated in the workshops. This sample size reflected the numbers attending the heart failure multidisciplinary team meetings and is in line with the Aspen Institute’s guide to Theory of Change development which recommends a maximum of eight to ten participants [30]. Three participants attended all the workshops, each with a different profession, which ensured continuity and maintained the focus of the discussions. The workshops were facilitated by BR with support from AG and NP, audio-recorded, and transcribed. The focus of the workshop discussions is presented in Table 1.

**Table 1:** Focus of the theory of change workshop discussions

Steps for developing the Theory of Change*	Description	Focus of which workshop
Identifying the impact of the intervention	The real-world change that stakeholders are trying to achieve.	**Workshop-1**,** Workshop-2**
Identifying the long-term outcomes	The final outcome that the intervention can achieve on its own.	**Workshop-2**,** Workshop-3**
Identifying the preconditions	Intermediate and short-term outcomes of the intervention that need to exist for the logical causal chain not to be broken.	**Workshop-2**,** Workshop-3**
Defining the interventions	The strategies and activities that should be done to achieve specific outcomes.	**Workshop-3**
Identifying the rationales	Key beliefs, based on scientific evidence or local experience, that underlie why an outcome is a precondition for the next, and why intervention activities must exist to produce desired outcomes.	**Workshop-2**,** Workshop-3**
Articulating the assumptions	External conditions beyond the control of the intervention that must exist to achieve the outcomes.	**Workshop-2**,** Workshop-3**
Ceiling of accountability	The level at which the intervention is no longer responsible for the target impact.	**Workshop-3**
Developing a pathway of change	Linking all the above elements in a map that shows how and why the complex intervention is expected to work (mechanism of action).	**All workshops**

* Based on the Aspen Institute’s and De Silva et al.’s practical guides to Theory of Change development [30, 31]

The nominal group technique was used in the first workshop to establish a consensus on the desired impact of the intervention [34]. Participants were given five minutes to generate ideas and asked to write their answers independently on provided cards. Next, each participant was asked to present one answer to the group in a round-robin fashion. This continued until no new suggestions were forthcoming. After that, the whole group discussed the answers and grouped similar ones. These groups were written on a flip chart where everyone could see them. In the second workshop, further discussions among participants resulted in an agreement on the desired impact of the intervention.

### Follow-up meetings with service providers

Three one-hour follow-up online meetings were conducted with two key service providers who participated in the Theory of Change workshops. The whole heart failure multidisciplinary team had been invited but as these meetings were conducted in July, September, and November 2021 during the COVID-19 pandemic, only two could attend. The aims were to discuss the secondary data analysis findings to refine the preliminary theory which was developed in the Theory of Change workshops, and to explore if and how the heart failure team’s clinical practice had been changed after the pandemic to evaluate what changes were necessary. The meetings were video-recorded and transcribed.

### Consultation with service users

After the follow-up meetings with service providers, a one-hour online consultation was conducted in January 2022 with patients from the Advanced Heart Failure James Lind Alliance to get feedback on the proposed intervention activities. The James Lind Alliance is a non-profit-making initiative, partly funded by the National Institute for Health Research, which ensures that people most affected by a condition are involved in prioritising research [35].

### Data analysis

The workshop discussions and analysis were informed by the Normalisation Process Theory constructs and components represented in the theory toolkit [36] (Additional File 2). Normalisation Process Theory is a theory that evaluates factors that affect the routine incorporation of a complex intervention into everyday life and thus helps to understand how and why a complex intervention works [37, 38].

The Theory of Change for this intervention was developed through an ongoing process of reflection and adaptation, allowing multiple iterations and modifications of the theory as appropriate. The content of the workshops’ and subsequent follow-up meetings’ transcripts were not formally analysed. Instead, it served to extract the Theory of Change elements that emerged from the discussions. Following the first workshop, BR collated and grouped the answers from the nominal group discussion; differentiated what could be an impact from what could be a long-term outcome or precondition; extracted possible intervention activities, preconditions, rationales, and assumptions from the discussions; added more components from the relevant literature; and linked them in Theory of Change map drafts.

The quality of the proposed Theory of Change was evaluated for its *plausibility* (do evidence and common sense suggest that the proposed activities will lead to the desired outcomes? ), *feasibility* (will the resources be available to carry out the initiative? ), and *testability* (is the proposed theory specific and complete enough for an evaluator to track its progress? ) [30, 31]. The Theory of Change approach for intervention development was reported using the Checklist for Reporting Theory of Change in Public Health Interventions [39], while the intervention itself was reported using the Template for Intervention Description and Replication (TIDieR) guidelines [21].

### Ethical considerations

Ethics approval for the group workshops was obtained from the Research Ethics Committee in the Faculty of Health and Medicine at Lancaster University on 9th August 2019 (reference number: FHMREC18098). Ethics approval for the follow-up meetings with service providers was obtained from the same committee on 20th July 2021 (reference number: FHMREC20170). Ethics approval was not required by the same Research Ethics Committee for the consultation with service users as this was a PPI group consultation that aimed to get feedback from service users on the proposed intervention.

## Results

### Participants in workshops

The demographic characteristics of the participants in the Theory of Change workshops are displayed in Table 2. Participants had an average duration of professional experience of 20 years. Most were females and heart failure nurse specialists from the acute hospital team. Three key participants attended all the workshops: a consultant cardiologist and heart failure specialist (team leader), lead heart failure nurse specialist, and heart failure occupational therapist. The workshops lasted for a median of 65 min.

**Table 2:** Demographic characteristics of the theory of change workshops’ participants

	Workshop-1	Workshop-2	Workshop-3	Total*
Participants	8	4	5	10
Female gender	7	4	4	8
Profession				
Consultant cardiologist	1	1	2	2
Heart failure nurse specialist (acute hospital team)	4	2	2	5
Heart failure nurse specialist (community team)	1	0	0	1
Heart failure occupational therapy specialist	1	1	1	1
Resident doctor	1	0	0	1
Years of professional experience: Mean (SD)	16.4 (7.0)	18.8 (5.3)	24.4 (4.8)	20.0 (8.6)

* As some people attended more than one workshop, the numbers do not add up

### Preliminary theory of change

The preliminary Theory of Change map constructed throughout the workshops is depicted in Additional File 3. The map shows the provisional intervention’s impact, long-term outcomes, preconditions, activities, assumptions, rationales, and hypothetical pathway of change. The impact of the intervention agreed upon was:

- Meeting the holistic palliative care needs of patients and families in a relevant timeframe.

Three long-term outcomes were proposed:

- Patients and families feel satisfied and supported.
- Primary palliative care needs of patients and families are addressed.
- Unnecessary hospitalisations are reduced.

While the intervention will contribute to meeting the palliative care needs of patients and families in a relevant timeframe (impact), it cannot achieve it solely on its own. This is known as the *ceiling of accountability* and sits between the long-term outcomes and the impact in the Theory of Change.

Twelve interconnected preconditions on the healthcare professional, patient and family, and organisational levels were identified to achieve the long-term outcomes. They were classified into three broad categories:

- Identification of patients with heart failure who have palliative care needs.
- Communication with patients and families and between healthcare professionals.
- Education of patients and families about progressive heart failure and healthcare professionals about palliative care.

In line with our systematic review findings [32], participants preferred to adopt the Needs Assessment Tool: Progressive Disease - Heart Failure (NAT: PD-HF) in their clinical practice to identify palliative care needs. Participants believed that this tool is more relevant to patients with heart failure compared to other tools and thought that its ‘Action Taken’ section would trigger them to act on the identified palliative care needs [40]. The main concerns on NAT: PD-HF were the difficulty of asking questions related to spiritual or existential issues and the lack of resources, skills, and time to identify and act on the identified needs. However, participants believed that with adequate training, these issues are surmountable.

The provisional intervention activities, rationales, and assumptions for each link in the causal pathway are detailed in Additional File 4. Nine intervention activities were proposed:

- NAT: PD-HF training.
- Using NAT: PD-HF in clinics.
- Acting on primary palliative care needs.
- Sharing experiences of using NAT: PD-HF.
- Writing NAT: PD-HF summaries in clinic letters.
- Sharing clinic letters with healthcare staff.
- Communicating and collaborating with healthcare staff, patients, and families.
- Educating patients and families on progressive heart failure.
- Signposting the heart failure team to palliative care training courses.

Ten rationales for the intervention activities and causal links in the Theory of Change map were identified. Where no rationale was found, assumptions exist instead. Assumptions were identified for six causal links in the Theory of Change map. Major assumptions were the availability of time, resources, skills, willingness, relationships, and information exchange systems. The lack of these contextual conditions may create barriers to achieving the long-term outcomes.

### Follow-up meetings with service providers

The meetings were attended by the hospital heart failure team leader and lead heart failure nurse specialist. Participants acknowledged the initial impact of the COVID-19 pandemic on the clinical practice of the heart failure team but assured that the proposed theory underpinning the intervention was still feasible. The findings of our secondary data analysis of the experiences of advanced heart failure patients, family carers, and healthcare professionals with palliative care services were presented and discussed against the findings of the Theory of Change workshops to refine the underlying theory [6]. For example, while the Theory of Change workshops concluded that patients and families should be educated about the progressive nature of heart failure, secondary data analysis findings showed that they also need information about medications, available care services and what they offer, and when to call for professional help in emergencies. This was discussed with participants who agreed that patient and family education should cover all the above items. Other examples of such discussions which contributed to refining the intervention are shown in Additional File 5.

### Consultation with service users

The PPI group agreed with the suggested intervention activities and supported targeting family carers in the intervention. They agreed that the intervention could be delivered to patients with heart failure not too early when the illness is stable, nor too late when the patient is dying. They also agreed that the intervention could benefit patients referred to heart failure and cardiology outpatient clinics for specialist review after hospital discharge. The group pointed to certain barriers that could impede the integration of palliative care in heart failure such as poor communication between clinicians, public misperception of palliative care, and the complexity of palliative care conversations with patients and families. Strategies to address these issues were considered to refine the complex intervention.

### Refined theory of change

After the follow-up meetings with key service providers and PPI group consultation, the preliminary Theory of Change developed through the group workshops was further refined. Long-term outcomes were refined based on feedback:

- Unnecessary hospitalisations are reduced.
- Patient symptom burden is reduced.
- Family caregiving burden is reduced.

**Fig. 2:**
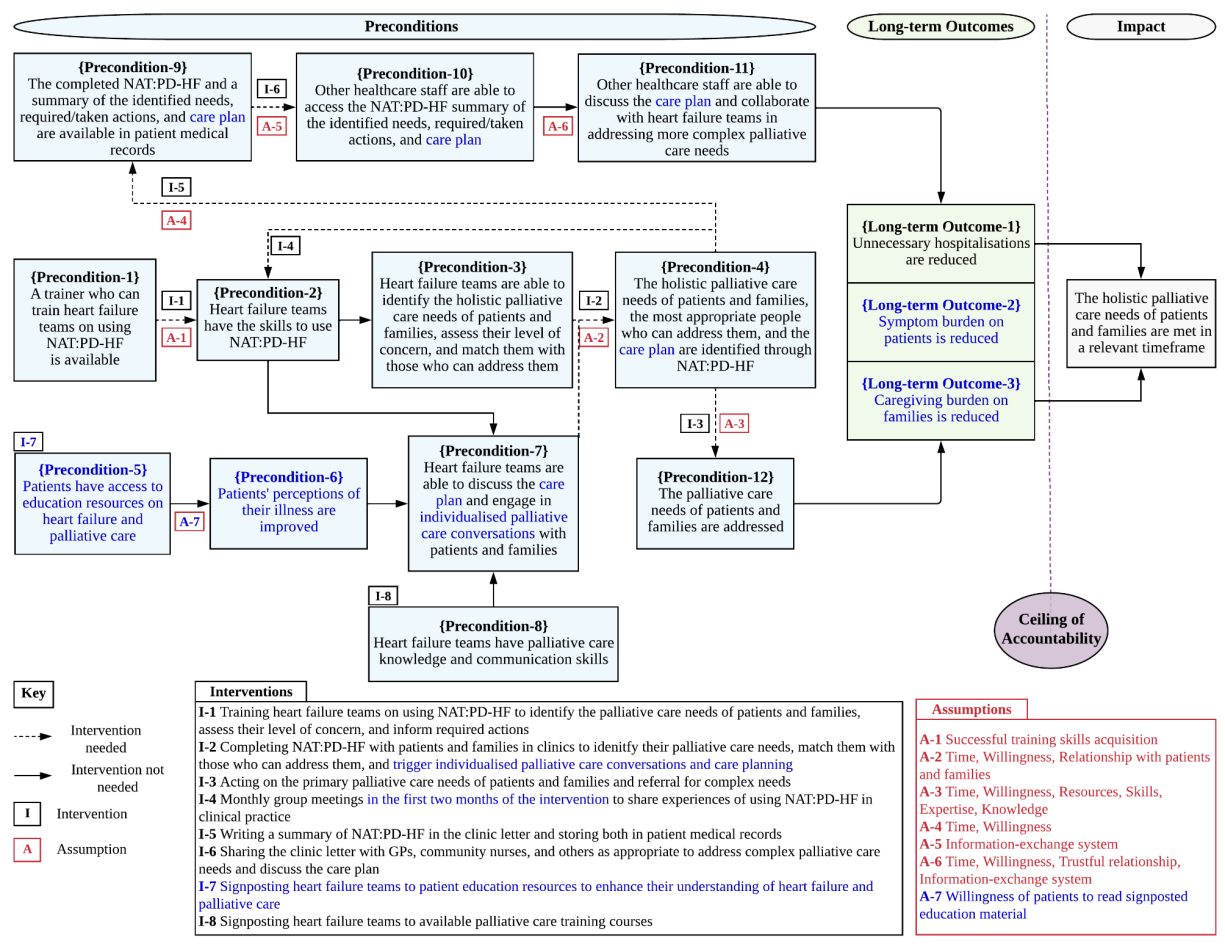
Refined theory of change map (changes written in blue colour)

A few preconditions and intervention activities were also refined, while the intervention impact, underlying assumptions and rationales, and hypothetical causal pathway of change stayed almost the same. The refined Theory of Change map is displayed in Fig. 2.

### Refined intervention

The modifications to the intervention based on feedback from service providers and users are summarised in Table 3. A systematic description of the refined intervention according to the TIDieR checklist is presented in Additional File 6. Briefly, the intervention will be delivered by hospital and community multidisciplinary heart failure teams. It will be provided to patients with heart failure referred to the hospital and community heart failure and cardiology outpatient clinics for a specialist review two weeks after hospital discharge. The intervention includes:

- A training component where heart failure teams will be trained on using NAT: PD-HF and signposted to palliative care training courses and patient education resources on heart failure and palliative care.
- Activities required by the heart failure team including completing NAT: PD-HF with patients and families to identify palliative care needs and trigger palliative care conversations, acting on primary palliative care needs, writing NAT: PD-HF summary, sharing the summary with healthcare staff, and conducting monthly meetings to share experiences of using NAT: PD-HF in clinical practice.

**Table 3:** Modifications to the preliminary intervention

Criteria	Preliminary intervention	Refined intervention
Intervention activities	Nine intervention activities.	Five intervention activities required by heart failure team staff, preceded by three training activities.
Education materials for patients and family carers	Undetermined.	British Heart Foundation booklets and an associated website; stickers of local health services and helpline numbers attached to the booklets.
Focus of patient education	Heart failure as a progressive disease.	More general (heart failure, available care services, who to call if condition deteriorates, palliative care); refer to educational resources.
Team’s willingness for advance care planning	Reluctance; favouring discussing the management plan instead.	More willingness; aided by NAT: PD-HF.
Staff responsible for palliative care conversations	Mostly not the heart failure team.	Mostly the heart failure team; refer patients for exceptional, complex conversations.
Open versus individualised conversations	Debated.	Individualised; looking actively for patient triggers.
NAT: PD-HF completion frequency	Monthly or with a change in patient condition.	Baseline, after six months, and with a change in patient condition.
NAT: PD-HF group meetings	Monthly throughout the intervention period.	Monthly in the first two months of the intervention.
Study settings where the intervention is delivered	Undetermined.	Hospital and community heart failure and cardiology outpatient clinics; all heart failure team members.
Target patient sample	All patients with heart failure attending the heart failure and cardiology outpatient clinics.	Patients with heart failure referred to heart failure and cardiology outpatient clinics for specialist review two weeks after hospital discharge.
Intervention duration	Undetermined; a few months.	Six months or until patient death, whichever is earlier.
Study outcomes to measure success of the intervention	Twelve preconditions and three long-term outcomes; undetermined outcome measures (or indicators in the Theory of Change terminology).	A few preconditions and long-term outcomes were refined; outcome measures were discussed and are subject to a future feasibility study.

## Discussion

Given the poor access of patients with heart failure to palliative care and the lack of guidance on palliative care integration, this study aimed to develop and refine a theory-based, complex palliative care intervention for patients with heart failure and their family carers. An important output is the co-development of a novel evidence-based theoretical framework for the intervention using the participatory Theory of Change approach to explain how it is expected to work in clinical practice. This was described using a narrative summary and presented graphically in a Theory of Change map that demonstrates the hypothetical causal pathway through which the intervention components interact to achieve specific long-term outcomes. The intervention activities, preconditions, assumptions, and rationales for each link in the causal pathway were identified, alongside the ultimate impact that the intervention would contribute to achieving.

The steps and theories that underpinned the intervention development were described in detail in this study. Such details were insufficiently reported in most other palliative care interventions for patients with heart failure, which makes it difficult to assess the thoroughness of the intervention components, conceptual frameworks, and mechanisms of action [39]. For this intervention, Normalisation Process Theory and the Theory of Change were used to consider implementation issues from the outset. Both theories have not been used before to develop a palliative care intervention for patients with heart failure. Theories used to inform the development of other palliative care interventions in heart failure pay less emphasis on implementation processes and what work should be done to integrate interventions into practice [41]; leading to simplistic, non-context-specific, and potentially less effective interventions [42, 43].

The desired impact of the proposed intervention was to meet the holistic palliative care needs of patients and families, which is one of the goals of palliative care according to the World Health Organisation [44]. The long-term outcomes were to reduce hospitalisations, symptom burden, and caregiving burden; all of which are common consequences of heart failure which need to be addressed in palliative care interventions [6]. Preconditions needed to achieve these outcomes were related to the identification of palliative care needs, communication, and education. This is comparable to the preconditions identified in similar palliative care studies using the Theory of Change approach [25–29] which may reflect a common understanding of the requirements of palliative care interventions despite different research focus and settings. Identified assumptions were related to the availability of time, resources, relationships, training skills acquisition, and commitment of all actors involved. These assumptions reflect some barriers to palliative care identified in the literature [16]. Addressing such barriers would enhance the delivery of and access to palliative care for patients with heart failure and contribute to meeting their palliative care needs.

The developed intervention comprises multiple intervention activities, reflecting the complex multi-component nature of palliative care [45]. While some activities are similar to those reported in other palliative care interventions [25–27], a few differences exist. One difference is the adoption of NAT: PD-HF to identify the holistic palliative care needs of patients and families as it showed superiority over other tools as demonstrated in our systematic review and after discussion with stakeholders in the Theory of Change workshops [32]. Other interventions either used criteria developed by the authors or adopted a less psychometrically robust tool to identify palliative care needs. Although NAT: PD-HF was a key component of Janssen et al.’s intervention, the non-culturally adapted Dutch translation rather than the original tool was used [46]. A novel intervention activity for this study is the use of NAT: PD-HF as both a palliative care needs-assessment tool and a sensitising tool to initiate and structure the palliative care conversations.

### Strengths and limitations

A strength of this study is the robust and iterative approach to developing the intervention through adopting complementary guiding frameworks and theories. Investment in the development stage helps overcome the ethical and practical difficulties in palliative care research [20]. The implementation of palliative care interventions in heart failure clinical practice is challenging [47]. A strength of this study is the participatory approach to developing the intervention, where stakeholders were involved throughout all stages and implementation issues were considered from the outset.

One issue is that the service providers involved in co-designing the intervention were from one site only, though they had worked in other heart failure units in the UK. Another issue is that only two service providers participated in the follow-up meetings to refine the proposed theory underpinning the intervention. In addition to the pandemic, this may be attributed to the long time elapsed between the group workshops, during which the preliminary theory was developed, and the follow-up meetings, during which the proposed theory was refined (about one and a half years). During this time, a secondary data analysis of the experiences with palliative care services was conducted and added useful insight to the intervention. If there were more participants in the follow-up meetings, the findings might have been different, as more views could have been explored. However, the two service providers who participated in the follow-up meetings were key members of the heart failure team who were involved in the intervention development from the outset and throughout the whole process of research, including the practitioner engagement meetings and shadowing activities before the workshops, the Theory of Change workshops, and subsequently the follow-up meetings. Besides, these members had a long duration of professional experience and held leadership positions (Cardiologist/heart failure team leader and Lead heart failure nurse specialist) and hence were expected to have an impact on the entire team.

General practitioners and palliative care specialists, who are typically involved in palliative care interventions for patients with heart failure, were not included in the group workshops. Including palliative care specialists in the workshops was discussed with the hospital heart failure team who preferred not to invite *outsider* practitioners at this stage of the intervention development. It was agreed that the intervention we were developing lies within the generalist palliative care domain, and one of the aims was to enhance the skills of the hospital heart failure team to address primary palliative care needs, while referring more complex needs to the palliative care specialists. Indeed, this was evident in the Theory of Change map in Precondition-11 and Intervention-6 (see Fig. 2). Still, the voice of palliative care specialists, as well as general practitioners, was included through the secondary data analysis which helped refine the theory underpinning the intervention after the group workshops (see Fig. 1).

Similarly, the service users were not included in the group workshops. While the initial focus was to develop a programme theory with service providers, patients and family carers would have been included at later stages in the Theory of Change workshops if it was not the COVID-19 pandemic. The Theory of Change components could have been different if patients and family carers had participated. However, the voice of patients and family carers was included from the secondary data analysis and PPI group consultation, which aided in refining the proposed Theory of Change after the workshops. Future studies should involve service users in the Theory of Change workshops.

The Theory of Change approach used in the intervention development comprised integrating the best current scientific evidence from research with stakeholder views. Although the developed Theory of Change could provide a model for similar palliative care interventions, it is not necessarily generalisable to different contexts nor applicable to other countries with different healthcare systems. Still, the clear pathway of change would enable researchers to assess the extent to which the preconditions, rationales, and assumptions are applicable in their context and evaluate whether the intervention components are transferrable or require adaptation. The developed Theory of Change could also apply to patients with other life-limiting illnesses. However, this needs further research and adaptations should be made such as using another needs-assessment tool as NAT: PD-HF is heart failure-specific.

### Implications for future research, practice, and policy

This study lays the foundation for further design and evaluation of a complex palliative care intervention for patients with heart failure in outpatient settings and their family carers to explore in more depth whether, how, and why the intervention works best in routine practice. The next step is to test the causal links and assumptions identified in the developed Theory of Change and evaluate the feasibility and acceptability of the intervention components for further adaptation to optimise the intervention before conducting a full trial. If these assumptions are met in the feasibility study, the proposed causal links will prove to be valid; and if not met, the intervention should be adjusted to fulfil the assumptions. Subsequently, the intervention effectiveness needs to be evaluated in a definitive randomised controlled trial with an embedded process evaluation, after which the intervention components and Theory of Change could further be refined before implementation in clinical practice.

The study adds to the recommendation of integrating multidisciplinary, team-based palliative care in the practice of heart failure teams to improve the care experiences of patients and families [10–13]. Heart failure teams are advised to adopt a holistic needs-based approach to palliative care [48]. Using the heart failure-specific NAT: PD-HF is strongly recommended to identify palliative care needs, triage actions to address such needs, and facilitate palliative care conversations [32]. The recommendations for education and training about the usefulness and usability of NAT: PD-HF can be transferable into a wide range of care settings supporting people with heart failure. This study may improve the confidence of health care practitioners in having timely conversations and initiating palliative care services for this patient population.

## Conclusions

This study presents novel insights into palliative care research in heart failure. It provides a systematic and participatory approach to developing complex palliative care interventions using a combination of complementary frameworks and theories. Through using a programme theory, the study provides a better understanding of the active ingredients and mechanism of integrating palliative care into standard heart failure care, which would enhance implementation potential. Meeting the holistic palliative care needs of patients and families is the desired impact of the proposed intervention. Reducing hospitalisations, symptom burden, and caregiving burden are the agreed long-term outcomes. Preconditions and intervention activities needed to achieve this goal are mainly related to the identification of palliative care needs, communication, and education. The main contextual assumptions are the availability of time, resources, commitment, relationships, and information exchange systems.

## Electronic supplementary material

Below is the link to the electronic supplementary material.

Supplementary Material 1

Supplementary Material 2

Supplementary Material 3

Supplementary Material 4

Supplementary Material 5

Supplementary Material 6

## Data Availability

The datasets analysed during the current study are available from the corresponding author on reasonable request.

## Declarations

### Declarations

All methods were performed in accordance with the relevant guidelines and regulations.

## Abbreviations

MORECare: Methods of Researching End of Life Care
MRC: Medical Research Council
NAT: PD-HF: Needs Assessment Tool: Progressive Disease - Heart Failure
PPI: Patient and Public Involvement
TIDieR: Template for Intervention Description and Replication
