# Supplementary material for: Development of a complex palliative care intervention for patients with heart failure and their family carers: a theory of change approach
Source: BMC Palliat Care. 2025 May 6;24:129. doi: 10.1186/s12904-025-01776-5 (PMC12057136; doi:10.1186/s12904-025-01776-5)
Supplement: Supplementary file 1 — Supplementary Material 1 [file 12904_2025_1776_MOESM1_ESM.docx]

Additional File 1: Steps of the intervention development

| Steps | Description |
| --- | --- |
| Identifying and defining the problem | Exploring the nature and impact of the problem of integrating palliative care into standard heart failure care in the research setting from different stakeholder perspectives to provide insights into the current gaps and investigate who is affected. |
| Examining current practice and context | Investigating the existing intervention practice and research setting to enhance the delivery and implementation of the intervention within its context and discussing the facilitators and barriers of the proposed intervention with stakeholders to adapt it to the current practice. |
| Determining service providers’ and users’ needs | Exploring the perceptions of stakeholders regarding the problem of palliative care integration, the palliative care needs of patients with heart failure and their families, and the preferences and capacities regarding the proposed intervention to produce a feasible and effective intervention. |
| Identifying the relevant existing evidence* | Identifying the relevant existing evidence by conducting a systematic review of palliative care needs-assessment and measurement tools in heart failure and finding systematic reviews of similar palliative care interventions to investigate their effectiveness. |
| Developing appropriate theory* | Developing a theory to understand the mechanism and process of the intervention (how it works and causes change and what the active ingredients are), identify possible barriers and reinforce weak links in the causal pathways, and subsequently create an effective, implementable, pragmatic, and sustainable intervention. |
| Modelling process and outcomes* | Refining the intervention and delineating the key intervention components in detail, and the mechanisms through which they interact to achieve specific outcomes, to allow comparisons and create an implementable, replicable, and transparent intervention. |
| Intervention design | Describing the output and final product of the development process including the final content, components, and duration of the intervention. |

* The main steps of the development stage in the MRC framework. The other steps and their description are adopted from Bleijenberg et al.’s guidance and MORECare statement.

Description of data: The steps of the complex intervention development based on the MRC framework, Bleijenberg et al.’s guidance, and MORECare guidance.
