# Supplementary material for: Development of a complex palliative care intervention for patients with heart failure and their family carers: a theory of change approach
Source: BMC Palliat Care. 2025 May 6;24:129. doi: 10.1186/s12904-025-01776-5 (PMC12057136; doi:10.1186/s12904-025-01776-5)
Supplement: Supplementary file 2 — Supplementary Material 2 [file 12904_2025_1776_MOESM2_ESM.docx]

Additional File 2: Application of the Normalisation Process Theory toolkit in informing and analysing the workshops

| Toolkit items* | Applications |
| --- | --- |
| Coherence | |
| Differentiation:  Participants distinguish the intervention from current ways of working. | The intervention was co-designed with service providers, described in detail, and broken down into individual intervention activities. Service providers could see how it is distinct from the routine heart failure care provided in their settings as it includes introducing new training materials, assessment tools, group meetings, and other activities. |
| Communal specification:  Participants collectively agree on the purpose of the intervention. | Service providers had a consensus and shared understanding of the intervention aims, expected outcomes, and desired impact; all of which were depicted in the Theory of Change map. |
| Individual specification:  Participants individually understand what the intervention requires of them. | Service providers understood how the intervention could affect the nature of their work. The specific tasks and responsibilities were explained to participants by breaking down the complex intervention into individual activities. |
| Internalisation:  Participants construct the potential value of the intervention for their work. | The intervention was developed in response to the service providers’ willingness to improve how they work with palliative patients. They asked for a service that enables better integration of palliative care into patients’ standard care. |
| Cognitive Participation | |
| Initiation:  Key individuals drive the intervention forward. | The intervention was championed by two key service providers who attended all the workshops and supplementary meetings. Both have a team leader position in their practice and are therefore able to support the intervention and get others involved. |
| Legitimation:  Participants agree that the intervention should be part of their work. | Service providers were aware that palliative care needs better integration in their practice and were willing to be involved in the new intervention. |
| Enrolment:  Participants' buy-in to the intervention. | Service providers were willing to adopt the intervention, open to working with colleagues in new ways, and prepared to challenge expected difficulties. |
| Activation:  Participants continue to support the intervention. | Service providers were willing to keep the intervention going by addressing the contextual conditions that may facilitate or impede the long-term survival of the intervention (time, resources, skills). |
| Collective Action | |
| Interactional workability:  Participants perform the tasks required by the intervention. | As service providers were involved in developing the intervention and discussing the contextual conditions, they believed they could integrate it into their existing work. |
| Relational integration:  Participants maintain their trust in each other’s work and expertise through the intervention. | Service providers had confidence in each other’s ability to use the intervention based on their knowledge, skills, and experience. They believed that the intervention would enhance their communication with each other, rather than disrupt their working relationships. |
| Skill set workability :  The work of the intervention is appropriately allocated to participants. | The intervention will be delivered by healthcare professionals with appropriate skills. Training will be provided to enhance participants’ skills in palliative care, communication, and use of needs-assessment tools. |
| Contextual integration:  The intervention is adequately supported by its host organisation. | The intervention was designed with service providers considering the available resources and support. Unfeasible intervention elements which were beyond the capacity of the available resources were excluded. |
| Reflexive Monitoring | |
| Systematisation:  Participants access information about the intervention effects. | Service providers will be provided with reports about the effects of the intervention after being evaluated. |
| Communal appraisal:  Participants collectively assess the intervention as worthwhile. | Service providers will be asked to provide their feedback on the intervention feasibility and its effect on their practice after being implemented. |
| Individual appraisal:  Participants individually assess the intervention as worthwhile. | Service providers will be asked to provide their feedback on the intervention feasibility and effect on their work after being implemented. |
| Reconfiguration:  Participants modify their work in response to their appraisal of the intervention. | Service providers’ feedback will be used to improve the intervention. |

* The items correspond to the Normalisation Process Theory sub-constructs.

Description of data: The application of the Normalisation Process Theory toolkit in informing and analysing the group workshops with service providers
