# Supplementary material for: Development of a complex palliative care intervention for patients with heart failure and their family carers: a theory of change approach
Source: BMC Palliat Care. 2025 May 6;24:129. doi: 10.1186/s12904-025-01776-5 (PMC12057136; doi:10.1186/s12904-025-01776-5)
Supplement: Supplementary file 3 — Supplementary Material 3 [file 12904_2025_1776_MOESM3_ESM.docx]

Additional File 3: Preliminary Theory of Change map


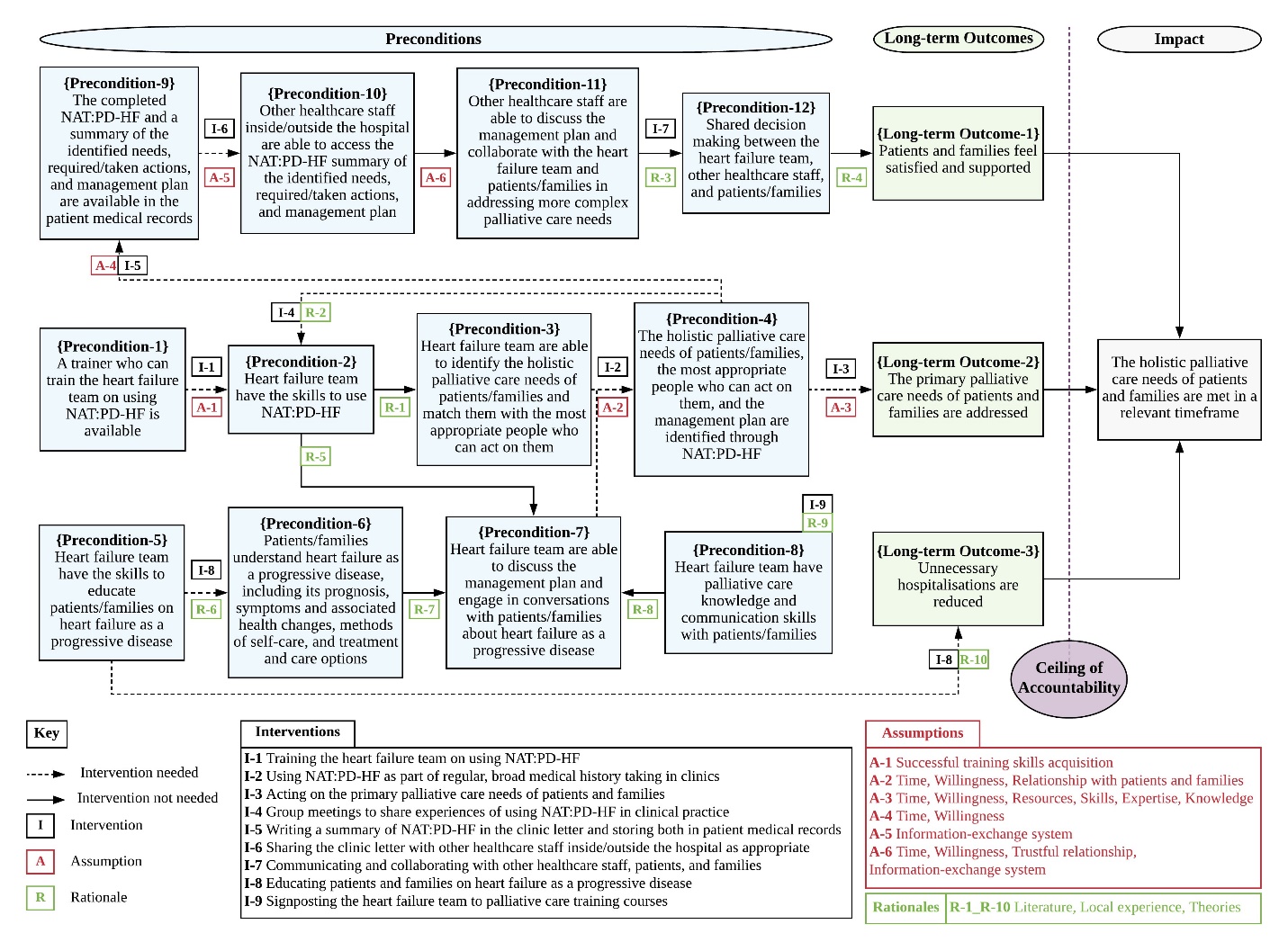


Description of data: The preliminary Theory of Change map underpinning the intervention developed through group workshops with service providers
