# Supplementary material for: Development of a complex palliative care intervention for patients with heart failure and their family carers: a theory of change approach
Source: BMC Palliat Care. 2025 May 6;24:129. doi: 10.1186/s12904-025-01776-5 (PMC12057136; doi:10.1186/s12904-025-01776-5)
Supplement: Supplementary file 4 — Supplementary Material 4 [file 12904_2025_1776_MOESM4_ESM.docx]

Additional File 4: Intervention activities, rationales, and assumptions underlying the preliminary Theory of Change

| Precondition-2: Heart failure team have the skills to use NAT:PD-HF | |
| --- | --- |
| Required Intervention | **[I-1]*** Training the heart failure team on how to use NAT:PD-HF to identify patient and family palliative care needs, assess their level of concern, and assign actions to address them. |
| Assumption | **[A-1]** The heart failure team should acquire the necessary skills from the NAT:PD-HF training session to use NAT:PD-HF effectively. |
|  | |
| Required Intervention | **[I-4]** Facilitated group meetings between heart failure team members to share experiences of using NAT:PD-HF in clinical practice. |
| Rationale for the intervention | **[R-2]** Participants suggested that group meetings following the use of NAT:PD-HF in clinics will help them to reflect on using the tool, learn more from each other, and resolve any issues. A systematic review showed that practice-based small group learning, which includes discussing patient cases, improves the knowledge and skills of learners and contributes to their professional development^347^. This approach is well-accepted by learners and can be adapted based on their learning needs^347^. Practice-based learning is supported by educational adult learning theories that indicate that individuals learn better and are more willing to change when they begin with problems that they have experienced in practice (problem-based learning)^348^. |
| Precondition-3: Heart failure team are able to identify the holistic palliative care needs of patients and families and match them with the most appropriate people who can act on them | |
| Required Intervention | no intervention required |
| Rationale for: Precondition-2 🡪  Precondition-3 | **[R-1]** Research shows that needs-assessment tools, like NAT:PD-HF (**Precondition-2**), can facilitate the timely recognition and holistic assessment of the palliative care needs of patients with heart failure^126,128,129,132,164^. The systematic review findings demonstrated some evidence that patients with heart failure who have palliative care needs can be identified by NAT:PD-HF^125^. When NAT:PD-HF was displayed to workshop participants, they were confident that it would prompt them to match the identified needs with the appropriate services to address them. |
| Precondition-4: The holistic palliative care needs of patients and families, the most appropriate people who can act on them, and the management plan are identified through NAT:PD-HF | |
| Required Intervention | **[I-2]** Using NAT:PD-HF as part of regular, broad medical history taking during patient consultation in the clinics of the heart failure team. |
| Assumption | **[A-2]** The heart failure team should have time, willingness, and good relationships with patients and families to use the tool during the clinic consultation. |
| Precondition-6: Patients and families understand heart failure as a progressive disease, including its prognosis, symptoms and associated health changes, methods of self-care, and treatment and care options | |
| Required Intervention | **[I-8]** Educating patients and families on heart failure as a progressive disease, including its prognosis, symptoms, self-management, and care options using existing educational materials and other resources. |
| Rationale for the intervention | **[R-6]** Educational interventions for patients with heart failure have been shown to improve their knowledge and understanding of the disease, medication, diet, and rationale for symptom monitoring^349,350^. Topics included in such education programmes were diagnosis and prognosis, pathophysiologic effect of heart failure, aims of treatment, management and symptom monitoring, medications and side effects, and prompts to call the GP. Service providers were confident about their ability to educate patients and families as they do this routinely in their practice. |
| Precondition-7: Heart failure team are able to discuss the management plan and engage in conversations with patients and families about heart failure as a progressive disease | |
| Required Intervention | no intervention required |
| Rationale for: Precondition-6 🡪  Precondition-7 | **[R-7]** The lack of knowledge on heart failure among patients and families (**Precondition-6**) is a well-documented barrier to palliative care communication^351^. A prospective study showed that patients with advanced illness who were educated about their illness, symptoms, and treatment had significantly better communication with healthcare professionals about their concerns^350^. A randomised controlled trial demonstrated a significantly higher quality of end-of-life communication between healthcare professionals and patients with heart failure who were educated about their disease^352^. |
|  | |
| Required Intervention | no intervention required |
| Rationale for: Precondition-2 🡪  Precondition-7 | **[R-5]** Palliative care needs-assessment tools (**Precondition-2**) have been shown to facilitate patient communication with healthcare professionals when completed by patients with heart failure before their clinic consultation^132^. NAT:PD-HF, although typically completed during the clinic consultation, has also been suggested to enhance the conversation between patients and the healthcare team^164^. Although the Dutch translation of NAT:PD-HF were not found helpful to communicate about palliative care, this was attributed to the lack of palliative care knowledge and communication skills among the staff rather than an issue with the tool itself^165^. |
|  | |
| Required Intervention | no intervention required |
| Rationale for: Precondition-8 🡪  Precondition-7 | **[R-8]** The lack of palliative care knowledge and communication skills among healthcare professionals (**Precondition-8**) is a well-documented barrier to palliative care communication with patients and families^165,353^. Systematic reviews showed that enhancing the communication skills of healthcare professionals was positively associated with the quality of communication with patients and can improve and increase palliative care discussions^354-356^. |
| Precondition-8: Heart failure team have palliative care knowledge and communication skills with patients and families | |
| Required Intervention | **[I-9]** Signposting heart failure team members to palliative care training courses such as communication skills and advance care planning. |
| Rationale for the intervention | **[R-9]** Systematic reviews provided evidence that training healthcare professionals on end-of-life communication, advance care planning, and palliative care in general improves their communication skills with patients about end-of-life issues^355,357-359^. Workshop participants were aware of some palliative care training programmes and showed a willingness to attend them. |
| Precondition-9: The completed NAT:PD-HF and a summary of the identified needs, required and taken actions, and management plan are available in the patient medical records | |
| Required Intervention | **[I-5]** Writing a summary of NAT:PD-HF in the clinic letter (including the identified needs, required and taken actions, and management plan), and storing NAT:PD-HF and the letter in patient medical records. |
| Assumption | **[A-4]** The heart failure team should have time and willingness to write a summary and store it, together with NAT:PD-HF, in patient records. |
| Precondition-10: Other healthcare staff inside and outside the hospital are able to access the NAT:PD-HF summary of the identified needs, required and taken actions, and management plan | |
| Required Intervention | **[I-6]** Sharing the NAT:PD-HF summary in the clinic letter with other healthcare professionals inside and outside the hospital as required. |
| Assumption | **[A-5]** An electronic or paper-based information-exchange system should exist to share the NAT:PD-HF summary with other healthcare staff. |
| Precondition-11: Other healthcare staff are able to discuss the management plan and collaborate with the heart failure team, patients, and families in addressing more complex palliative care needs | |
| Required Intervention | no intervention required |
| Assumption | **[A-6]** An information-exchange system should exist to facilitate communication between the heart failure team and other healthcare staff. Time, willingness, and trustful relationships are needed to collaborate in patient care. |
| Precondition-12: Shared decision making between the heart failure team, other healthcare staff, patients, and families | |
| Required Intervention | **[I-7]** Communication and collaboration with other healthcare staff inside and outside the hospital, patients, and families. |
| Rationale for the intervention | **[R-3]** The lack of multidisciplinary communication and trustful relationship was perceived by patients and families as a major barrier to shared decision making^360,361^. Communication interventions aimed at healthcare professionals were shown to improve shared decision making with patients^362,363^. The Conceptual Framework for Individual and Family End-of-Life Decision Making indicates that shared decision making is facilitated through an interdisciplinary team approach and by enhancing information sharing between patients, families, and healthcare providers^364^. |
| Long-term outcome-1: Patients and families feel satisfied and supported | |
| Required Intervention | no intervention required |
| Rationale for:  Precondition-12  🡪  Long-term outcome-1 | **[R-4]** Two systematic reviews showed that patient and family involvement in decision making (**Precondition-12**) is associated with their satisfaction with palliative care^365,366^. For patients with heart failure, those who had shared decision making had high *satisfaction with decision* scores and felt supported^367^. |
| Long-term outcome-2: The primary palliative care needs of patients and families are addressed | |
| Required Intervention | **[I-3]** Acting on the primary palliative care needs of patients and families identified by the heart failure team. |
| Assumption | **[A-3]** To address the palliative care needs of patients and families, the heart failure team members should have time, willingness, resources, and necessary skills, expertise, and knowledge. |
| Long-term outcome-3: Unnecessary hospitalisations are reduced | |
| Required Intervention | **[I-8]** Educating patients and families on heart failure as a progressive disease, including its prognosis, symptoms, self-management, and care options using existing educational materials and other resources. |
| Rationale for the intervention | **[R-10]** Systematic reviews showed that educational interventions for patients with heart failure and their families significantly reduce hospitalisation and rehospitalisation^368-370^. |

* The numbers given to the preconditions, interventions, assumptions, and rationales are concordant with those in the preliminary Theory of Change map in **Additional File 3.**

Description of data: Intervention activities, rationales, and assumptions underlying the preliminary Theory of Change for the proposed intervention following the group workshops with service providers
