# Supplementary material for: Development of a complex palliative care intervention for patients with heart failure and their family carers: a theory of change approach
Source: BMC Palliat Care. 2025 May 6;24:129. doi: 10.1186/s12904-025-01776-5 (PMC12057136; doi:10.1186/s12904-025-01776-5)
Supplement: Supplementary file 5 — Supplementary Material 5 [file 12904_2025_1776_MOESM5_ESM.docx]

Additional File 5: Findings of the Theory of Change workshops against findings of the secondary data analysis

* Discussing relevant findings from Theme-1 (Impact of heart failure)

| Findings from Theory of Change workshops | Finding from secondary data analysis |
| --- | --- |
| Heart failure team use NAT:PD-HF to assess the palliative care needs of patients and families. | Patients and families had multidimensional palliative care needs: physical, psychological, social, spiritual, practical, medication issues, and information needs. |
|  | |
| Heart failure team educate patients and families about heart failure as a progressive disease, including prognosis, symptoms, methods of self-care, and care options. | Patients and families also needed information about medications; available care services and what they can offer (including palliative care services); whether, when, and whom to call for professional help in illness and emergencies; and financial issues.  Patients and families wanted the use of lay language in education. |

* Discussing relevant findings from Theme-2 (Coping and support)

| Findings from Theory of Change workshops | Finding from secondary data analysis |
| --- | --- |
| Heart failure team communicate and collaborate with other healthcare staff inside and outside the hospital. | Multiplicity of healthcare professionals caused GPs to take a backseat and made it difficult to collaborate, communicate, share information, and coordinate care. |
|  | |
| Heart failure team act on the primary palliative care needs of patients and families. | Most healthcare professionals did not adopt a holistic, patient-centred palliative care approach. |

* Discussing relevant findings from Theme-3 (Recognising palliative phase)

| Findings from Theory of Change workshops | Finding from secondary data analysis |
| --- | --- |
| Heart failure team are able to discuss the management plan and engage in conversations with patients and families about heart failure as a progressive disease (reluctance to engage in open advance care plan discussions). | Most patients, families, and professional caregivers favoured open conversations about diagnosis, prognosis, and end of life. Although open conversations could cause initial worry to patients and families or could be difficult due to prognostic uncertainty and poor understanding of heart failure and palliative care, they enable discussing patient wishes and care plans and prompt patients to reflect, confront fears, and make decisions in their life. Some healthcare professionals favoured individualised conversations that are led by patients for what, how much, and when to have information. |
|  | |
| The intervention should be delivered to all patients attending the hospital, and perhaps community, heart failure clinics. | Early palliative care and advance care planning build relationships, make patients more prepared for conversations, reduce the chance of missing health services, address palliative care needs, and improve quality of life. |

* Discussing relevant findings from Theme-4 (Coordination of care)

| Findings from Theory of Change workshops | Finding from secondary data analysis |
| --- | --- |
| Other healthcare staff inside and outside the hospital are able to communicate and collaborate with the heart failure team, patients, and families.  The heart failure team share the NAT:PD-HF summary in the clinic letter with other healthcare staff as appropriate. | Poor communication and information sharing between healthcare professionals and with patients and families. |
|  | |
| Heart failure team are signposted to available palliative care training courses.  Heart failure team conduct group meetings to share experiences of using NAT:PD-HF in practice.  Heart failure team are informed of the available local healthcare services for referrals as part of the NAT:PD-HF training. | Collaborative education is important including joint training, joint visits to patients’ homes, shadowing, and multidisciplinary palliative care meetings.  Healthcare professionals needed information about the available care services and their role as this would help in role clarification, avoiding conflicts, seeking advice, and offering referrals to address patient and family care needs. |
|  | |
| Heart failure team store the NAT:PD-HF summary and management plan in the clinic letter and share it with other healthcare staff using an appropriate information-exchange system. | A common practice was to store medical documents in patients’ homes in an accessible place so that other staff can see them. |
|  | |
| Heart failure team complete NAT:PD-HF monthly or with a change in patient condition (functional status). | Patients need continuous follow-up and monitoring. |
|  | |
| Heart failure team aim to meet the palliative care needs of patients and families in a relevant timeframe. | Patients need a timely response to their care needs. |
|  | |
| Trustful relationships between healthcare professionals and with patients and families are important contextual factors. | Staff consistency improves relationships, communication, and continuity of care. |

Description of data: Findings of the Theory of Change workshops with service providers against findings of the secondary data analysis about the experiences of patients, family carers, and healthcare professionals with palliative care services
