# Supplementary material for: Development of a complex palliative care intervention for patients with heart failure and their family carers: a theory of change approach
Source: BMC Palliat Care. 2025 May 6;24:129. doi: 10.1186/s12904-025-01776-5 (PMC12057136; doi:10.1186/s12904-025-01776-5)
Supplement: Supplementary file 6 — Supplementary Material 6 [file 12904_2025_1776_MOESM6_ESM.docx]

Additional File 6: Systematic description of the refined intervention according to the TIDieR checklist

| Item | Description |
| --- | --- |
| Name | A theory-based, complex palliative care intervention for patients with heart failure and their family carers. |
|  | |
| Who? | The intervention will be provided by hospital and community multidisciplinary heart failure teams. |
|  | |
| What ‘procedures’?  How? | **Training session for the heart failure teams:**   - Training heart failure teams on using NAT:PD-HF to identify the palliative care needs of patients and families, assess their level of concern, and inform required actions in a one-hour, in-person or online interactive group training session per each study centre. - Signposting heart failure teams to available palliative care training courses at the end of the session. - Signposting heart failure teams to patient education resources on heart failure and palliative care at the end of the session. |
|  | **Intervention activities required by the heart failure teams:**   - Completing NAT:PD-HF with patients and families in clinics at baseline, after six months, and with change in patient functional condition to identify their palliative care needs, match them with those who can address them, and trigger individualised palliative care conversations and care planning. - Acting on the primary palliative care needs of patients and families based on clinical expertise and referral for more complex needs. - Writing a summary of the identified needs, required actions, and care plan for each completed NAT:PD-HF in the clinic letter, and storing NAT:PD-HF and the clinic letter in patient medical records. - Sharing the clinic letter with GPs, community nurses, and others as appropriate using available information-exchange systems to address complex palliative care needs and discuss the care plan. - Monthly one-hour in-person or online interactive group meetings in the first two months of the intervention to share experiences of using NAT:PD-HF in clinical practice. |
|  | |
| Why? | - The intervention was developed in response to the poor and late access of patients with heart failure to palliative care and the lack of guidance on how to integrate palliative care into standard heart failure care. - The overall goal (impact) of the intervention is to meet the holistic palliative care needs of patients and families in a relevant timeframe. - The rationales for the intervention components are outlined in **Additional File 3**. |
|  | |
| What ‘materials’? | - Training material on using NAT:PD-HF for heart failure teams. - List of available palliative care training courses for heart failure teams. - Patient education resources (two British Heart Foundation booklets, an associated website, and a list of local healthcare services) to enhance their understanding of heart failure and palliative care. - NAT:PD-HF to identify the palliative care needs of patients and families, assess their level of concern, match the needs with those who can address them, and trigger palliative care conversations and care planning. - Clinic letters to record a summary of NAT:PD-HF. - Information-exchange systems to share the NAT:PD-HF summary, refer patients and families to healthcare services, and communicate with other staff. - Other materials as required to act on the primary palliative care needs of patients and families, such as medications and information sheets. |
|  | |
| Where? | The intervention will be delivered in hospital and community heart failure and cardiology outpatient clinics in the UK. |
|  | |
| When?  How much? | - The intervention will be provided to patients with heart failure referred to the heart failure and cardiology outpatient clinics for a specialist review two weeks after hospital discharge. - In the first study period, the intervention will be delivered only to patients in the fast-track group; at a later point of time (12 weeks), it will also be delivered to patients in the standard control group. - The intervention will be delivered over six months per patient or until patient death, whichever is earlier. |
|  | |
| Tailoring | To meet the individual needs of heart failure teams in different settings and enhance the intervention feasibility and implementation, flexibility and tailoring of the intervention would be allowed without compromising its core components; for example, the teams could use any suitable information-exchange system to share the NAT:PD-HF summary with other staff, but they should not use a different needs-assessment tool. |
|  | |
| How well? | The plan for assessing the intervention adherence and fidelity is subject to a future feasibility study. |

Description of data: Systematic description of the refined palliative care intervention according to the TIDieR guidelines
